# Elevated branched-chain amino acid promotes atherosclerosis progression by enhancing mitochondrial-to-nuclear H_2_O_2_-disulfide HMGB1 in macrophages

**DOI:** 10.1016/j.redox.2023.102696

**Published:** 2023-04-05

**Authors:** Shuai Zhao, Lei Zhou, Qin Wang, Jia-Hao Cao, Yan Chen, Wei Wang, Bo-Da Zhu, Zhi-Hong Wei, Rong Li, Cong-Ye Li, Geng-Yao Zhou, Zhi-Jun Tan, He-Ping Zhou, Cheng-Xiang Li, Hao-Kao Gao, Xu-Jun Qin, Kun Lian

**Affiliations:** a Department of Cardiology, Xijing Hospital, The Fourth Military Medical University, Xi'an, Shaanxi, 710032, China; b Department of Clinical Laboratory Medicine, Xijing Hospital, The Fourth Military Medical University, Xi'an, Shaanxi, 710032, China; c Department of Pharmacogenomics, The Fourth Military Medical University, Xi'an, Shaanxi, 710032, China; d School of Public Health, Shaanxi University of Chinese Medicine, Xianyang, Shaanxi, 712046, China; e Department of Cardiology, No.971 Hospital of the PLA Navy, Qingdao, Shandong, 266071, China; f Primary Flight Training Base, Air Force Aviation University, Harbin, Heilongjiang, 150100, China; g Department of Geriatrics, Xijing Hospital, The Fourth Military Medical University, Xi'an, Shaanxi, 710032, China; h Department of Nutrition and Food Hygiene, School of Public Health, The Fourth Military Medical University, Xi'an, Shaanxi, 710032, China; i Department of Health Statistics, Ministry of Education Key Lab of Hazard Assessment and Control in Special Operational Environment, School of Public Health, The Fourth Military Medical University, Xi'an, Shaanxi, 710032, China; j Department of Cardiovascular Surgery, The First Affiliated Hospital of Xi'an Jiaotong University, Xi'an, Shaanxi, 710061, China; k Key Laboratory for Space Biosciences and Biotechnology, School of Life Sciences, Northwestern Polytechnical University, Xi'an, Shaanxi, 710072, China

**Keywords:** Atherosclerosis (AS), Branched-chain amino acid (BCAA), Macrophage, Inflammation, Hydrogen peroxide (H_2_O_2_), Mitochondria, HMGB1

## Abstract

As the essential amino acids, branched-chain amino acid (BCAA) from diets is indispensable for health. BCAA supplementation is often recommended for patients with consumptive diseases or healthy people who exercise regularly. Latest studies and ours reported that elevated BCAA level was positively correlated with metabolic syndrome, diabetes, thrombosis and heart failure. However, the adverse effect of BCAA in atherosclerosis (AS) and its underlying mechanism remain unknown. Here, we found elevated plasma BCAA level was an independent risk factor for CHD patients by a human cohort study. By employing the HCD-fed ApoE^−/−^ mice of AS model, ingestion of BCAA significantly increased plaque volume, instability and inflammation in AS. Elevated BCAA due to high dietary BCAA intake or BCAA catabolic defects promoted AS progression. Furthermore, BCAA catabolic defects were found in the monocytes of patients with CHD and abdominal macrophages in AS mice. Improvement of BCAA catabolism in macrophages alleviated AS burden in mice. The protein screening assay revealed HMGB1 as a potential molecular target of BCAA in activating proinflammatory macrophages. Excessive BCAA induced the formation and secretion of disulfide HMGB1 as well as subsequent inflammatory cascade of macrophages in a mitochondrial-nuclear H_2_O_2_ dependent manner. Scavenging nuclear H_2_O_2_ by overexpression of nucleus-targeting catalase (nCAT) effectively inhibited BCAA-induced inflammation in macrophages. All of the results above illustrate that elevated BCAA promotes AS progression by inducing redox-regulated HMGB1 translocation and further proinflammatory macrophage activation. Our findings provide novel insights into the role of animo acids as the daily dietary nutrients in AS development, and also suggest that restricting excessive dietary BCAA consuming and promoting BCAA catabolism may serve as promising strategies to alleviate and prevent AS and its subsequent CHD.

## Graphical abstract

**Figure:**
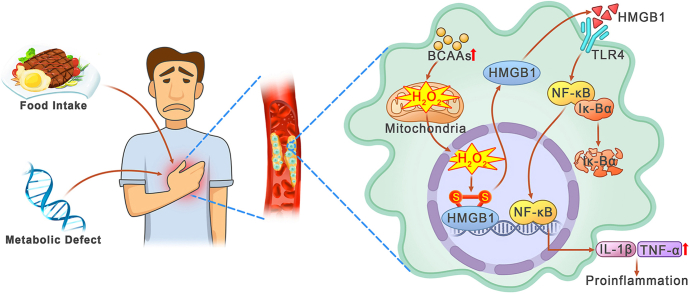

## Abbreviations:

8-OHdG: 8-hydroxy-2 deoxyguanosine
α-SMA: α-smooth muscle actin
ApoE^−/−^: apolipoprotein E-deficient
ALT: alanine transaminase
AST: aspartate aminotransferase
AS: atherosclerosis
AUC: area under the receiver operating curve
BCAA: branched-chain amino acid
BCAT: branched-chain amino-transferase
BCKA: branched-chain alpha-keto acid
BCKDH: branched-chain alpha-ketoacid dehydrogenase complex
BCKDHA: E1a subunit of BCAA dehydrogenase
BDK: branched-chain alpha-ketoacid dehydrogenase complex kinase
BMI: body mass index
BT2: 3,6-dichlorobenzo[b]thiophene-2-carboxylic acid
BUA: blood uric acid
CHD: coronary atherosclerotic heart disease
CI: confidence interval
DBP: diastolic blood pressure
EV: empty vector
FBG: fasting blood glucose
HCD: high cholesterol diet
HDL-C: high density lipoprotein cholesterol
HMGB1: high mobility group box 1
HMGB1-KD: high mobility group box 1 knock down
H_2_O_2_: hydrogen peroxide
KIC: α-ketoisocaproic acid
KIV: α-ketoisovaleric acid
KMV: α-keto-β-methylvaleric acid
LDL-C: low density lipoprotein cholesterol
MDA: malondialdehyde
mCAT: mitochondria-targeted catalase
mtH_2_O_2_: mitochondrial hydrogen peroxide
nCAT: nucleus-targeted catalase
p-BCKDHA: phosphorylated BCKDHA
PP2Cm: protein phosphatase 2Cm
SBP: systolic blood pressure
TC: total cholesterol
TG: triglyceride
WBC: white blood cell
WT: wild-type

## 1 Introduction

Branched-chain amino acid (BCAA), including leucine, isoleucine and valine, accounts for approximately 35% of the essential amino acids. Physiologically, as fundamentals building blocks of our body, BCAA is critical to maintaining intestinal health, immune function, energy supplement, nutrient metabolism, protein synthesis [1]. BCAA is often supplemented to mitigate cachexia, attenuate fatigue during exercise and improve wound healing by promoting anabolic pathways [2]. In contrast to these health-promoting effects, recent literature and our previous study found that elevated BCAA was positively correlated with metabolic syndrome, diabetes, thrombosis and heart failure [[3], [4], [5], [6]]. Atherosclerosis (AS) is responsible for many major adverse cardiovascular events, including atherosclerotic coronary heart disease (CHD), ischemic stroke and peripheral artery disease, covering most cardiovascular morbidity and mortality [7]. Multiple risk factors have been largely demonstrated associated with the development of AS, including smoking, hypertension, diabetes mellitus, obesity, and dyslipidemia [8]. Recently, Yang et al. reported that plasma BCAA was positively correlated with coronary artery disease [9]. However, how BCAA pathogenetically affects AS remains unknown.

AS is a chronic inflammatory disease that occurs within the arterial wall. During AS development, circulating monocytes attach to the surface of the arterial endothelium and infiltrate the aortic intima, where they differentiate into macrophages and further transform into foam cells with the accumulation of lipids [10]. During this process, the activation of macrophages is the key to initiating an inflammatory program to produce proinflammatory cytokines, contributing to AS progression [[11], [12], [13]]. Interestingly, Zhenyukh et al. reported that elevated BCAA could increase inflammation in endothelial cells via activating peripheral blood mononuclear cells [14,15]. Whether elevated BCAA is associated with atherosclerotic inflammation mediated by macrophages remains unclear, and the underlying molecular mechanisms are completely unknown.

In the present study, we found that elevated BCAA level was an independent risk factor in patients with CHD, and it promoted AS progression in mice. BCAA catabolic defects were observed in AS human monocytes and mouse macrophages, while increasing BCAA catabolism in macrophages ameliorated the AS progression. Elevated BCAA activated proinflammatory macrophages by increasing mitochondrial H_2_O_2_ (mtH_2_O_2_). Specifically, BCAA increased the formation and secretion of disulfide HMGB1 by mitochondrial-to-nuclear H_2_O_2_ signaling, thus activating TLR4/NF-κB pathway and downstream inflammatory cascades. These findings demonstrated that elevated BCAA independently contributed to the pathogenesis of AS, and also suggested that restricting excessive dietary BCAA intake and promoting BCAA catabolism may serve as promising strategies to alleviate and prevent AS and its subsequent CHD.

## 2 Materials and methods

### 2.1 Human participants

A total of 239 male patients with CHD hospitalized in the Department of Cardiology of Xijing Hospital (Xi'an, China) were consecutively enrolled from June 2018 to January 2020. Inclusion criteria: 1) The CHD patients were diagnosed by cardiac angiography, which was defined as diameter stenosis 50% for left main coronary artery and 70% for non-LM coronary artery within a vessel diameter ≥2.5 mm [16]; 2) The patients were ≥18 years of age and ≤75 years of age; 3) The patients were willing to provide the written informed consent. Exclusion criteria: 1) The patients were unwilling to undergo cardiac angiography; 2) The patients suffered from a history of heart failure, diabetes mellitus, peripheral artery disease, uncontrolled hypertension, acute myocardial infarction, or iodine contrast agent allergy; 3) The patients were at high risk of bleeding diathesis or coagulation disorders (e.g., malignancy, anemia, infectious disease or severe pulmonary disease, severe ventricular arrhythmias or clear hemodynamic fluctuations). 188 healthy males who received routine physiological examinations at Xijing Hospital between February 2019 and December 2019 were enrolled as healthy controls. Inclusion criteria: 1) The healthy controls were males and ≥18 years of age as well as ≤ 75 years of age; 2) The males were willing to provide the written informed consent. The healthy controls were excluded if they combined with heart failure, diabetes mellitus, peripheral artery disease, hypertension, or myocardial infarction. Venous blood samples were collected following overnight fasting before medication treatment. The blood samples were divided into two aliquots: one for monocyte isolation and the other for plasma preparation. This study was approved by the Ethics Committee of Fourth Military Medical University (KY20172019-1, Xi'an, China). Written informed consent was obtained from each participant.

### 2.2 Animals

This study was approved by the Fourth Military Medical University Committee on Animal Care (IACUC-20190150). All experiments were performed according to the National Institutes of Health Guidelines on the Use of Laboratory Animals. 8-week-old wild-type (WT) C57BL/6 mice and 8-week-old apolipoprotein E-deficient (ApoE^−/−^) C57BL/6 mice were obtained from the animal center of the Fourth Military Medical University. All mice were housed in a specific-pathogen-free environment with a 12/12 h light/dark cycle at 22–26 °C and 40–60% humidity.

### 2.3 Establishment of the AS model and drug treatment

All ApoE^−/−^ mice were fed with a high cholesterol diet containing 1.25% cholesterol (Research Diets, China) for 14 weeks to induce AS [17]. The ApoE^−/−^ mice were randomly divided into three groups: AS group, AS+3,6-dichlorobenzo[b]thiophene-2-carboxylic acid (BT2) group and AS + BCAA group. BT2, a BDK inhibitor that increases BCKDHA activity, and thus accelerates BCAA catabolism and decreases BCAA, was diluted in 5% DMSO, 10% cremophor EL and 85% of 0.1 mol/L sodium bicarbonate, pH 9.0. The mice in AS + BT2 group were intraperitoneally injected with BT2 at 40 mg/kg/day for 2 weeks when the mice were 20 weeks old, and the mice in other groups were given the same volume of vehicle. The mice in AS + BCAA group were fed with 3 mmol/L BCAA administered in drinking water. All the mice were free access to diet and water. The food intake were measured once weekly. After 14 weeks feeding of a high cholesterol diet for ApoE^−/−^ mice or a standard chow diet for C57BL/6 mice, the body weight was measured. The BCAA and BT2 were purchased from Sigma-Aldrich (USA) and US Biological (USA) respectively.

### 2.4 Bone marrow transplantation

Eight-week-old WT C57BL/6 mice were used as donors of bone marrow. The tibias and femurs of the four limbs of each donor were collected after mice were sacrificed. The bone cavity was rinsed with sterile PBS with a 1 mL syringe. The cells were collected by centrifugation after filtration through a 70-mesh filter. After centrifugation in 1 mL red blood cell lysate for 30 s, primary bone marrow cells were obtained. Cells were then divided into two aliquots, each containing 2 × 10^6^ cells. The cells were transduced with empty viral vectors or BCKDHA-overexpression viral vectors at a MOI of 15. Polybrene (4 μg/mL) was used to enhance infection efficiency. After a 10-min incubation, cells were centrifuged at 800 g for 30 min. The supernatant was discarded, and the cells were resuspended in sterile PBS. Eight-week-old ApoE^−/−^ mice were used as recipient mice and given sterile water containing gentamicin (320,000 U/L) and erythromycin (250 mg/L) 1 week before irradiation. After being lethally irradiated with 1000 rads (10 Gy) from a cesium source for 4 h, the mice were anesthetized with isoflurane and implanted with 200 μL bone marrow cell suspension containing 2 × 10^6^ cells via the jugular vein, and were subsequently subjected to an HCD diet intervention for 14 weeks [17].

### 2.5 Preparation of histopathological specimens

After the mice were euthanized, ice-cold PBS was slowly injected into the left ventricular apex to flush the blood vessels, and this was followed by perfusion fixation with 4% paraformaldehyde. Under gross microscopy, the aortas were separated, and the aortic root tissue was immediately embedded in optimal cutting temperature compound (Servicebio, China) at −20 °C. Continuous frozen sections of 10 μm thickness were prepared.

### 2.6 Oil red O staining

The slices were reheated and fixed in the fixative solution for 15 min. Next, the slices were stained with Oil Red solution (Servicebio, China) for 10 min in the dark. Then, the slices were immersed in two cup of 60% isopropanol for differentiation in turn, 3 s and 5 s respectively. Subsequently, the slices were re-stained with hematoxylin (Servicebio, China) for 1 min and rinsed with running water. Finally, the slices were sealed with glycerin gelatin and observed under a Nikon ECLIPSE E100 microscope. The individuals who quantified the microscopy were blinded.

### 2.7 Masson's trichrome staining

The slices were dewaxed and incubated in Masson A overnight. After being rinsed with tap water, the slices were stained with Masson B and Masson C in equal volumes for 1 min. Subsequently, the slices were differentiated with 1% hydrochloric acid alcohol, then incubated with Masson D for 6 min. Following the 1 min incubation with Masson E, the slices were stained with Masson F for 30 s, and then subjected to differentiation in 1% glacial acetic acid and dehydration with absolute ethanol and xylene. Finally, the slices were sealed with neutral gum (Sinopharm Chemical ReagentCo., Ltd, China) and observed under a Nikon ECLIPSE E100 microscope. The Masson dyes were from Servicebio (China). The individuals who quantified the microscopy were blinded.

### 2.8 Immunofluorescence staining

The slices were dewaxed and placed in a repair box filled with EDTA antigen repair buffer (pH 8.0). Antigen repair was performed in a microwave oven at medium power for 8 min and low power for 7 min at an 8-min interval. After the slices were slightly dried, 3% BSA was added to cover the tissue and incubated for 30 min at room temperature. The sealing solution was then gently removed. The slices were incubated with primary antibodies (F4/80, Novus, USA, 1:100; iNOS, Novus, USA, 1:100; α-SMA, Goldbio, USA, 1:800; TNF-α, Servicebio, China, 1:200) in a humid chamber at 4 °C overnight. After being washed three times, the slices were incubated with secondary antibodies (FITC-goat anti-rabbit, Goldbio, USA, 1:300; Cy3-goat anti-rabbit, Goldbio, USA, 1:300) at room temperature for 50 min in the dark. The nuclei were stained with DAPI (Goldbio, USA). After sealing with an anti-fluorescence quenching sealing tablet, the slices were observed, and images were acquired under an inverted Nikon Eclipse TI-SR fluorescence microscope. The individuals who quantified the microscopy were blinded.

### 2.9 Isolation of human blood monocytes and mouse peritoneal macrophages

Human blood monocytes were isolated with a Dynabeads FlowComp human CD14 kit (Thermo Fisher Scientific, USA) according to the manufacturer's instructions. Briefly, whole blood samples were incubated with FlowComp human antibody to CD14 for 10 min at 4 °C, then centrifuged in isolation buffer for 15 min at 350 g at 4 °C. The pellet was incubated with FlowComp Dynabeads under rolling and tilting for 15 min at 4 °C. Subsequently, the sample was mixed with isolation buffer and placed on a magnet for 3 min. The supernatant containing the CD14^−^ cells was removed. After the washing steps were repeated three times, the bead-bound CD14^+^ cells were resuspended in 1 mL FlowComp release buffer and incubated under rolling and tilting for 10 min at 4 °C. Finally, the beads were washed according to the manufacturer's protocol, and the bead-free CD14^+^ cells were resuspended in the medium and stored at 4 °C until use.

To isolate mouse peritoneal macrophages, the mice were intraperitoneally injected with 2 mL of 3% thioglycolate. 4 days later, the mice were euthanized and intraperitoneally injected with sterile PBS. The abdominal cavity was rinsed with PBS repeatedly with a syringe to collect macrophages.

### 2.10 Cell culture of RAW 264.7 cells and lentivirus transinfection

RAW 264.7 cells were obtained from the American Type Culture Collection and cultured in customized BCAA-free DMEM/HG supplemented with 10% FBS, 1% penicillin/streptomycin and certain concentration of exogenous BCAA as previously described by our lab [18,19]. As BCAA is essential amino acid, the DMEM medium supplemented with 0.4 mM exogenous BCAA was used for control, while the DMEM medium with 4 mM exogenous BCAA was used for BCAA treatment. The cells were treated with BCAA for 12 h before the samples were collected for further assay. The lentiviral vectors expressing scrambled shRNAs, mouse BCKDHA-shRNA, or HMGB1-shRNA were obtained from Orbitalgene (China). The sequences of BCKDHA-shRNA or HMGB1-shRNA were as follows: BCKDHA-shRNA (NM_007533.5): 5′-CCGGATTGTGATCTGTTACTT-3′, HMGB1-shRNA (NM_001313894.1) and 5′-CCCAGATGCTTCAGTCAACTTA-3'. Lentiviral vectors for overexpression of mouse catalase (CAT; NM_009804.2) with a mitochondrial targeting sequence (mCAT), mouse CAT with a nuclear targeting sequence (nCAT) and mouse BCKDHA were purchased from Orbitalgene. The empty vectors were used as negative controls. RAW 264.7 cells were infected with lentivirus. Cells with stable overexpression were selected with puromycin.

### 2.11 Liquid chromatography-tandem mass spectrometry (LC-MS)

The human plasma BCAA and BCKA were measured by Hangzhou Calibra Diagnostics (China) with isotope dilution LC-MS as previously described [20]. Standard l-leucine, l-isoleucine, l-valine, α-ketoisovaleric acid, α-keto-β-methylvaleric acid, α-ketoisocaproic acid, L-^13^C_1_-leucine and salicylic acid were purchased from Sigma-Aldrich. In brief, the plasma samples were pretreated with protein precipitation. LC-MS was performed with an Agilent Zorbax SB-C18 column and Sciex TripleQuad 4500MD. The animal plasma BCAA and cellular BCAA were measured with a BCAA detection kit (Biovision, USA) according to the manufacturer's instructions. The animal plasma BCKA and cellular BCKA were determined by HPLC as previously described [21].

### 2.12 Two-photon fluorogenic probe assays

MtH_2_O_2_ levels were measured with a mitochondria-targeted two-photon fluorogenic probe as aforementioned [22]. Cells were incubated with 10 μmol/L Mito-LX for 1 h and then stained with MitoTracker Red for 15 min. The cells were washed with PBS. Mito-LX fluorescence emission intensities were measured at 610 nm wavelength after excitation at 405 nm. Fluorescence images of mtH_2_O_2_ in live RAW264.7 cells were captured by LSM800 confocal laser scanning microscope (Carl Zeiss, Germany).

### 2.13 Flow cytometry

Flow cytometry was used to measure mtH_2_O_2_ levels and detect macrophage markers. For mtH_2_O_2_ measurement, cells were incubated with 10 μmol/L Mito-LX for 1 h and then collected. After cells were washed with PBS, mtH_2_O_2_ fluorescence intensity was measured with a Sony SA3800 flow cytometer (Sony, Japan). At least 20,000 events were recorded for each sample. The results were analyzed in FlowJo V10 software. Macrophages were incubated with antibodies to CD11C (Biolegend, USA) or CD206 (Biolegend, USA), so as to detect the macrophage markers. After incubated with the antibodies for 30 min, the macrophages were washed in PBS. Marker expression was analyzed by a BD LSRII flow cytometer (BD, USA).

### 2.14 Measurement of nuclear H_2_O_2_ levels with fluorescent HyPer

H_2_O_2_-sensitive fluorescent HyPer with a nuclear targeting sequence (Nu-HyPer) was obtained from Tsingke Biotechnology (China). Cells were infected with adenovirus (Hanbio, China) expressing Nu-Hyper. For live cell imaging, cells expressing Nu-Hyper were seeded in a confocal dish and cultured for 24 h before fluorescence imaging. Live cell images were captured with a confocal laser scanning microscope (Carl Zeiss, Germany) with a CFP-YFP dual filter (excitation, 420 and 500 nm; emission, 520 nm).

### 2.15 Antibody array assays

Differentially expressed proteins in macrophages exposed to BCAA were identified with antibody array assays by using a L-series mouse antibody array L-2 kit (RayBiotech, USA) according to the manufacturer's instructions. The fluorescein-labeled array was visualized with an InnoScan 300 microarray scanner. Data were collected in GenePix Pro 5.1 software and analyzed in RayBiotech Q-Analyzer software.

### 2.16 Quantitative real-time PCR (qRT-PCR)

Total RNA was isolated with TRIzol reagent (Ambion, USA) in compliance with the manufacturer's instructions. After the cDNA was synthesized, PCR was performed with a SYBR q-PCR kit (Vazyme, USA). GAPDH or β-actin was used as an internal reference. The sequences of primers are summarized in Table S2.

### 2.17 Western blot analysis

Cells were lysed with RIPA lysis buffer. Cytoplasmic and nuclear proteins were isolated with a protein extraction kit (KeyGEN BioTECH, China) according to the manufacturer's instructions. The quantity of protein was measured with a BCA protein assay kit (KeyGEN BioTECH). Protein (10–20 μg) was separated with SDS-PAGE and transferred to nitrocellulose membranes. Non-specific binding sites were blocked with 5% nonfat milk for 2 h, and blots were incubated overnight with primary antibodies to BCKDHA (Cell Signaling Technology, USA), p-BCKDHA (Bethyl Laboratories, USA), HMGB1 (Abcam, UK), p65 (Abcam, UK), IκBα (Cell Signaling Technology, USA), TBP (Abcam, UK), Tubulin (Bioworld Technology, USA) or GAPDH (Bioworld Technology, USA). After being rinsed with PBST, the membrane was incubated with secondary antibodies for 1 h at 37 °C. The protein bands were visualized with an enhanced chemiluminescence reagent (Millipore-Sigma, USA) on a Quantity One system (Bio-Rad, USA).

### 2.18 Enzyme-linked immunosorbent assay (ELISA)

Specific ELISA kits were used to measure IL-1β, TNF-α, and HMGB1 (Jianglai, China). The levels of the oxidative stress markers malondialdehyde (MDA) and 8-hydroxy-2 deoxyguanosine (8-OHdG) were measured with corresponding kits from Beyotime (China) and Jianglai according to the manufacturer's instructions.

### 2.19 Statistical analysis

Statistical analysis was performed by GraphPad Prism (version 8.0.1) and MedCalc (version 20.0.22) software. Data are expressed as mean ± standard deviation or mean ± standard error. Comparisons between two groups were performed with Student's *t*-test or the Wilcoxon rank-sum test. Comparisons among more than two groups were performed with one-way ANOVA and post hoc test by the Tukey's method. Logistic regression analysis was carried out for univariate and multivariate analysis. Receiver operating characteristic (ROC) curve analysis was performed to assess the performance of BCAA to distinguish the healthy controls from patients with CHD. A *P* value < 0.05 was considered statistically different.

## 3 Results

### 3.1 Elevated plasma BCAA level is an independent risk factor for CHD

**Fig. 1:**
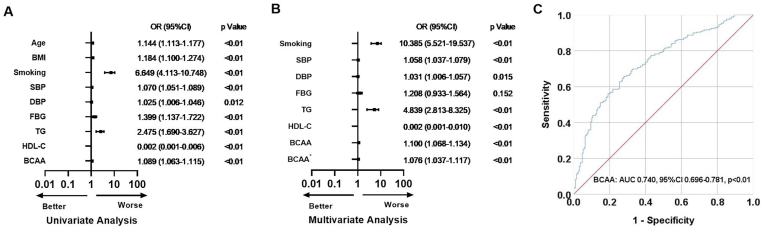
Elevated plasma BCAA level is an independent risk factor for CHD (A) Univariate logistic regression analysis for discrimination of CHD. (B) Multivariate logistic regression analysis for discrimination of CHD. (C) Areas under the receiver operating curve of BCAA for discrimination of CHD. BMI, body mass index; SBP, systolic blood pressure; DBP, diastolic blood pressure; FBG, fasting blood glucose; TG, triglyceride; HDL-C, high density lipoprotein cholesterol; BCAA, branched-chain amino acid. *Age, BMI, TG, TC, LDL-C, HDL-C and BCAA were included in multivariate logistic regression analysis.

CHD is prominently an atherosclerotic disease, with the highest annual morbidity and mortality [7]. A total of 446 men were consecutively enrolled in the current study, including 188 healthy volunteers and 258 patients with CHD who were diagnosed by cardiac angiography. The levels of the three components of BCAA, leucine, isoleucine and valine, and their respective ketoacid metabolites (branched-chain a-keto acid, BCKA), KIC, KMV and KIV in CHD patients were significantly higher than those in healthy controls (Table 1). Meanwhile, BCAA level was not correlated with glycolipid levels in CHD patients (Table S1). Plasma BCAA level was found to be an independent risk factor for CHD (OR: 1.076; 95%CI: 1.037–1.117; *P* < 0.01) after adjusting for age, BMI, FBG, TC, TG, LDL-C, and HDL-C (Fig. 1A and B), with the three individual BCAA, leucine, isoleucine and valine, correlated with CHD respectively, and the valine was the most important risk factor for CHD (Table S2). Furthermore, ROC analysis indicated that BCAA (AUC = 0.740, 95%CI: 0.696–0.781, *P* < 0.01) distinguished healthy volunteers from CHD patients (Fig. 1C). The optimal cutoff value of BCAA was 63.96 μg/mL, with 65.27% sensitivity and 72.34% specificity, suggesting that men with plasma BCAA level higher than 63.96 μg/mL were more likely to have CHD (Table 2).

**Table 1:** Baseline characteristics.

	Ctrl	CHD	χ^2^/t	p Value
n	188	239		
Male (%)	188 (100%)	239 (100%)		
Age (y)	48.16 ± 6.83	59.55 ± 10.90	44.899	<0.01
BMI (kg/m^2^)	23.55 ± 2.81	25.04 ± 3.31	0.529	<0.01
SBP (mm Hg)	115.41 ± 10.68	128.37 ± 16.56	23.641	<0.01
DBP (mm Hg)	71.81 ± 8.26	74.48 ± 11.72	21.524	0.007
Heart rate	66.18 ± 8.86	73.75 ± 9.57	0.034	<0.01
WBC (10^9/L)	6.02 ± 1.62	6.71 ± 1.97	6.129	<0.01
PLT (10^9/L)	208.87 ± 51.51	200.58 ± 57.73	0.328	0.126
Hb (g/L)	156.02 ± 8.96	146.20 ± 17.61	29.215	<0.01
ALT (IU/L)	24.51 ± 9.23	30.41 ± 22.33	20.945	<0.01
AST (IU/L)	21.84 ± 4.74	26.13 ± 25.72	15.544	0.013
SCr (μmol/L)	73.74 ± 8.82	82.79 ± 29.47	23.076	<0.01
BUA (μmol/L)	341.75 ± 53.20	333.49 ± 84.00	19.253	0.240
TC (mmol/L)	4.30 ± 0.52	3.43 ± 1.07	47.181	<0.01
TG (mmol/L)	1.17 ± 0.41	1.49 ± 0.81	25.391	<0.01
LDL-C (mmol/L)	2.64 ± 0.47	1.98 ± 0.97	34.561	<0.01
HDL-C (mmol/L)	1.30 ± 0.26	0.96 ± 0.21	5.688	<0.01
FBG (mmol/L)	4.97 ± 0.45	5.33 ± 1.44	58.867	<0.01
BCAA (μg/mL)	60.23 ± 9.75	70.62 ± 17.15	14.752	<0.01
Isoleucine (μg/mL)	11.15 ± 2.20	13.03 ± 3.60	18.219	<0.01
Leucine (μg/mL)	20.51 ± 3.23	23.47 ± 5.70	18.504	<0.01
Valine (μg/mL)	28.58 ± 4.81	34.12 ± 8.51	12.661	<0.01
BCKA (μg/mL)	9.19 ± 1.95	12.29 ± 3.25	16.454	<0.01
KIC (μg/mL)	4.56 ± 1.12	6.30 ± 1.72	12.559	<0.01
KMV (μg/mL)	2.54 ± 0.58	3.40 ± 0.91	13.586	<0.01
KIV (μg/mL)	2.09 ± 0.40	2.59 ± 0.73	16.571	<0.01
Medications at discharge, n%				
Aspirin		212 (88.70)		
P2Y12 inhibitors		202 (84.52)		
Statin		237 (99.16)		
ACE inhibitors or ARB		178 (74.48)		
β-Blockers		217 (90.79)		

BMI, body mass index; SBP, systolic blood pressure; DBP, diastolic blood pressure; WBC, white blood cell; PLT, platelet; Hb, hemoglobin; ALT, alanine transaminase; AST, aspartate Aminotransferase; SCr, serum creatinine; BUA, blood uric acid; TC, total cholesterol; TG, triglyceride; LDL-C, low density lipoprotein cholesterol; HDL-C, high density lipoprotein cholesterol; FBG, fasting blood glucose; BCAA, branched-chain amino acid; BCKA, branched-chain alpha-keto acid; KIC, α-ketoisocaproic acid; KMV, α-keto-β-methylvaleric acid; KIV, α-ketoisovaleric acid; ACE, angiotensin-converting enzyme; ARB, angiotensin receptor blockers.

**Table 2:** The ROC curve analysis of the BCAA with CHD.

	AUC	95%CI	p Value	Se (%)	Sp (%)	YI	Cutoff point
BCAA	0.740	0.696–0.781	<0.01	65.27	72.34	0.38	63.96

ROC, receiver operating characteristic; AUC, area under the receiver operating curve; CI, confidence interval; Se, sensitivity; Sp, specificity; YI, youden index; BCAA, branched-chain amino acid.

### 3.2 Elevated BCAA promotes AS progression in HCD-fed ApoE^−/−^ mice

**Fig. 2:**
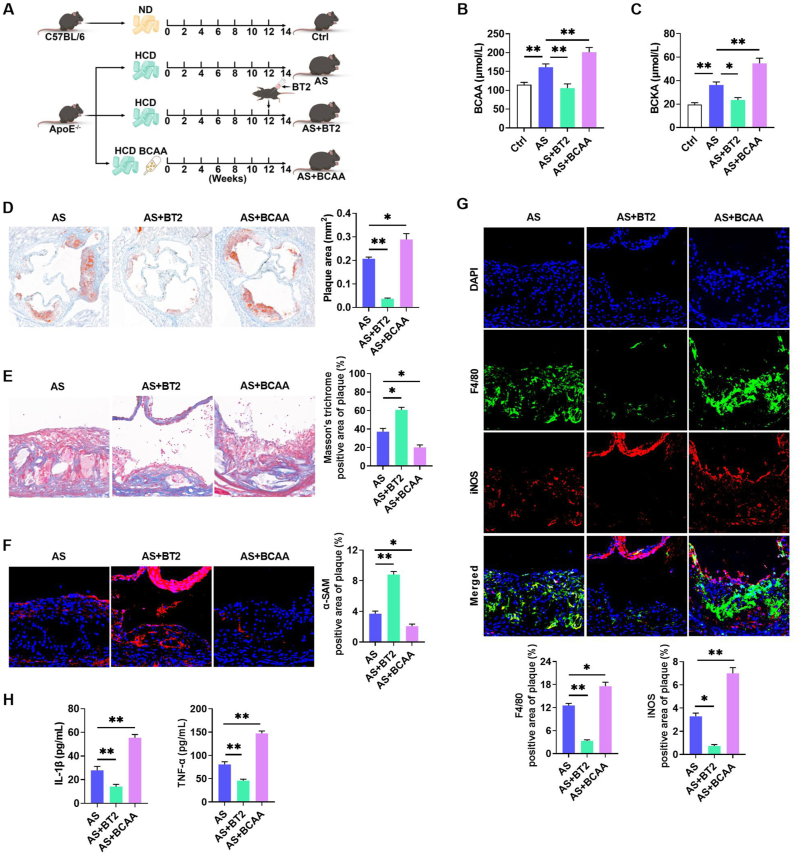
Elevated BCAA enhances AS progression in HCD-fed apoE^−/−^ mice (A) Schematic diagram of animal study. (B) Plasma BCAA level in mice. (C) Plasma BCKA level in mice. (D) Oil Red O staining of the plaque area. (E) Masson's trichrome staining of the collagen content. (F) Alpha-smooth muscle actin (α-SMA) positive cells by immunofluorescence staining showing the smooth muscle cells (SMCs). (G) F4/80 and iNOS positive cells by immunofluorescence staining showing macrophages. (H) Serum IL-1β and TNF-α levels in mice. Data were expressed as mean ± SEM, n = 5. All data were analyzed with one-way ANOVA followed by Tukey's multiple comparisons test. *P < 0.05; **P < 0.01. (For interpretation of the references to colour in this figure legend, the reader is referred to the Web version of this article.)

The homeostasis of BCAA is determined largely by the catabolic activities apart from the dietary intake. BCAA is catabolized to generate corresponding BCKA, which is irreversibly decarboxylated by the branched-chain a-keto acid dehydrogenase (BCKDH) in mitochondria [23]. The activity of BCKDH is regulated by the reversible phosphorylation of its E1a subunit (BCKDHA) by a specific kinase (BDK) and phosphatase (PP2Cm). BDK phosphorylates BCKDHA to inhibit its activity and result in the accumulation of BCAA and BCKA [1]. Additionally, BT2, as a BDK inhibitor, increases BCKDH activity while decreases BCAA and BCKA. To identify the role of BCAA in AS, we decreased or increased BCAA level by treating HCD-fed ApoE^**−/−**^ mice with either BT2 or exogenous BCAA (Fig. 2A). Plasma BCAA and BCKA levels were significantly higher in AS mice than those in WT mice, as shown in Fig. 2B and C. BT2 treatment markedly activated BCKDHA in liver (Fig. S2) consistent with other studies [24,25], demonstrating the validity of BT2 treatment as liver is the most important organ of BCAA metabolism. At the same time, BT2 treatment significantly decreased plasma BCAA and BCKA levels, whereas BCAA intake further elevated plasma BCAA and BCKA levels compared with AS mice (Fig. 2B and C). No significant change was observed in body weight, plasma glucose or lipid level in response to BT2 or BCAA intervention (Figs. S1A–F). Importantly, BT2 treatment significantly decreased plaque volume and increased plaque stability (increased collagen content and smooth muscle cell numbers) in the aortic root, while extra BCAA intake further promoted plaques progression (Fig. 2D–F). Given that AS is a chronic inflammatory disease characterized by the activation of macrophages [10], we measured plasma inflammatory cytokines and detected the proinflammatory macrophage (F4/80 and iNOS positive cells) in the plaques [26]. BT2 treatment decreased plasma IL-1β and TNF-α levels as well as F4/80 and iNOS expression, whereas extra BCAA intake further increased plasma inflammatory cytokines levels and proinflammatory macrophages in the plaques (Fig. 2G and H). These findings indicated that elevated BCAA significantly promoted AS progression and inflammation in HCD-fed ApoE^**−/−**^ mice.

### 3.3 Elevated BCAA activates proinflammatory macrophages

**Fig. 3:**
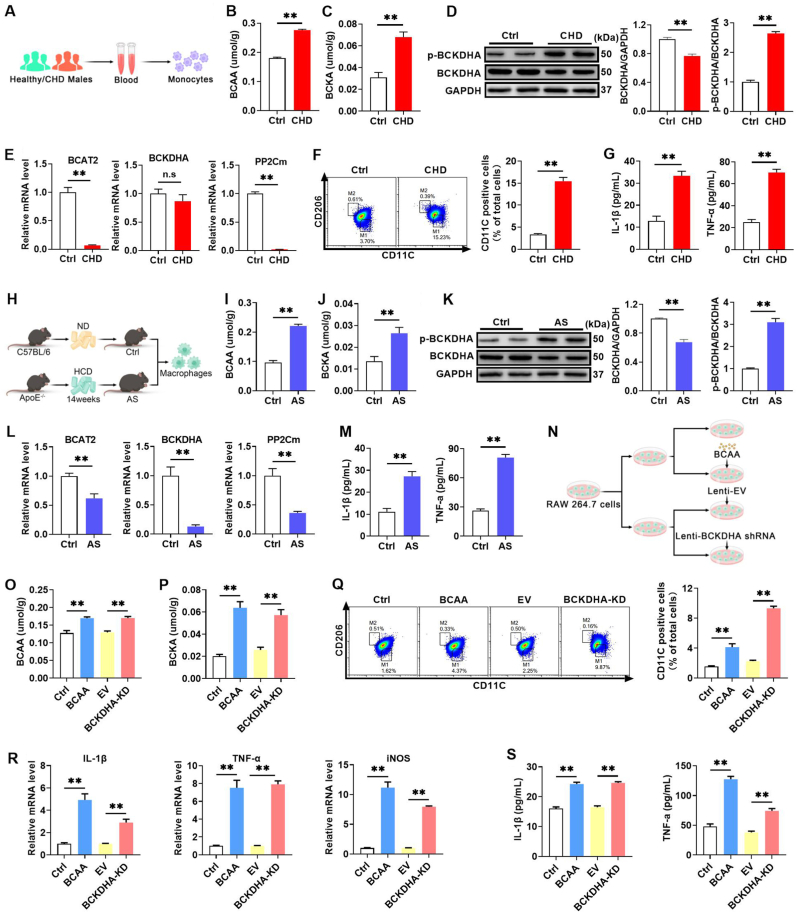
Elevated BCAA activates proinflammatory macrophages (A) Schematic diagram of experimental procedure for human circulating monocytes. (B) BCAA level in CHD patients' circulating monocytes. (C) BCKA level in CHD patients' circulating monocytes. (D) Protein levels of BCKDHA and p-BCKDHA/BCKDHA in CHD patients' circulating monocytes. (E) Expression levels of BCAA catabolism-promoting genes in CHD patients' circulating monocytes. (F) CD11C positive cells in CHD patients' circulating monocytes. (G) IL-1β and TNF-α levels in CHD patients' circulating monocytes. (H) Schematic diagram of experimental procedure for mice abdominal macrophages. (I) BCAA level in HCD-fed ApoE^−/−^ mice’ abdominal macrophages. (J) BCKA level in HCD-fed ApoE^−/−^ mice’ abdominal macrophages. (K) Protein levels of BCKDHA and p-BCKDHA/BCKDHA in HCD-fed ApoE^−/−^ mice’ abdominal macrophages. (L) Expression levels of BCAA catabolism-promoting genes in HCD-fed ApoE^−/−^ mice’ abdominal macrophages. (M) IL-1β and TNF-α levels in HCD-fed ApoE^−/−^ mice’ abdominal macrophages. (N) Schematic diagram of experimental procedure for RAW 264.7 macrophages (O) BCAA level in BCKDHA-KD RAW 264.7 macrophages. (P) BCKA level in BCKDHA-KD RAW 264.7 macrophages. (Q) CD11C positive cells in BCKDHA-KD RAW 264.7 macrophages. (R) Expression levels of the proinflammatory macrophage marker in BCKDHA-KD RAW 264.7 macrophages. (S) IL-1β and TNF-α levels in BCKDHA-KD RAW 264.7 macrophages. Data were expressed as mean ± SEM of three independent experiments. B–M: data were analyzed with unpaired Student *t*-test; O–S: data were analyzed with one-way ANOVA followed by Tukey's multiple comparisons test. **P < 0.01; n.s, not significant.

Macrophages are found in atherosclerotic arteries at all stages of the disease, from lesion initiation to plaque rupture, which can be activated to promote vascular inflammation and lesion development [27]. To clarify the role of BCAA in proinflammatory macrophage activation in AS development, we isolated monocytes from CHD patients and macrophages from AS mice (Fig. 3A). Notably, we found that BCAA and BCKA levels in patient monocytes were significantly higher than those in healthy controls (Fig. 3B and C), accompanied by substantial deactivation of BCKDHA (Fig. 3D) and decrease of BCAT2 and PP2Cm expressions (Fig. 3E). These findings suggested that the increased BCAA and BCKA in CHD patients' monocytes may be due to BCAA catabolic defects. It is worthy to note that the total BCKDHA expression decreased in CHD patients’ monocytes, providing a potential mechanism of BCAA catabolic defects for our future study. Moreover, CHD patients displayed a markedly higher percentage of CD11C positive proinflammatory macrophages [28] and higher levels of IL-1β and TNF-α, compared with the healthy controls (Fig. 3F and G). Similar results were observed in the abdominal macrophages from AS mice (Fig. 3H-M). Furthermore, we knocked down BCKDHA (BKKDHA-KD) expression to induce BCAA catabolic defects in mouse RAW 264.7 macrophages (Fig. 3N, Figs. S3A and S3B). Both BCAA supplementation and BCKDHA-KD caused accumulations of BCAA and BCKA in macrophages, along with substantial increase of the percentage of CD11C positive cells, as well as the expression and secretion of proinflammatory cytokines (Fig. 3O–S). Together, these findings suggested that elevated BCAA due to exogenous supplementation or endogenous catabolic defects induced activation of proinflammatory macrophages.

### 3.4 Mitochondrial H_2_O_2_ mediates BCAA-activated proinflammatory macrophages

**Fig. 4:**
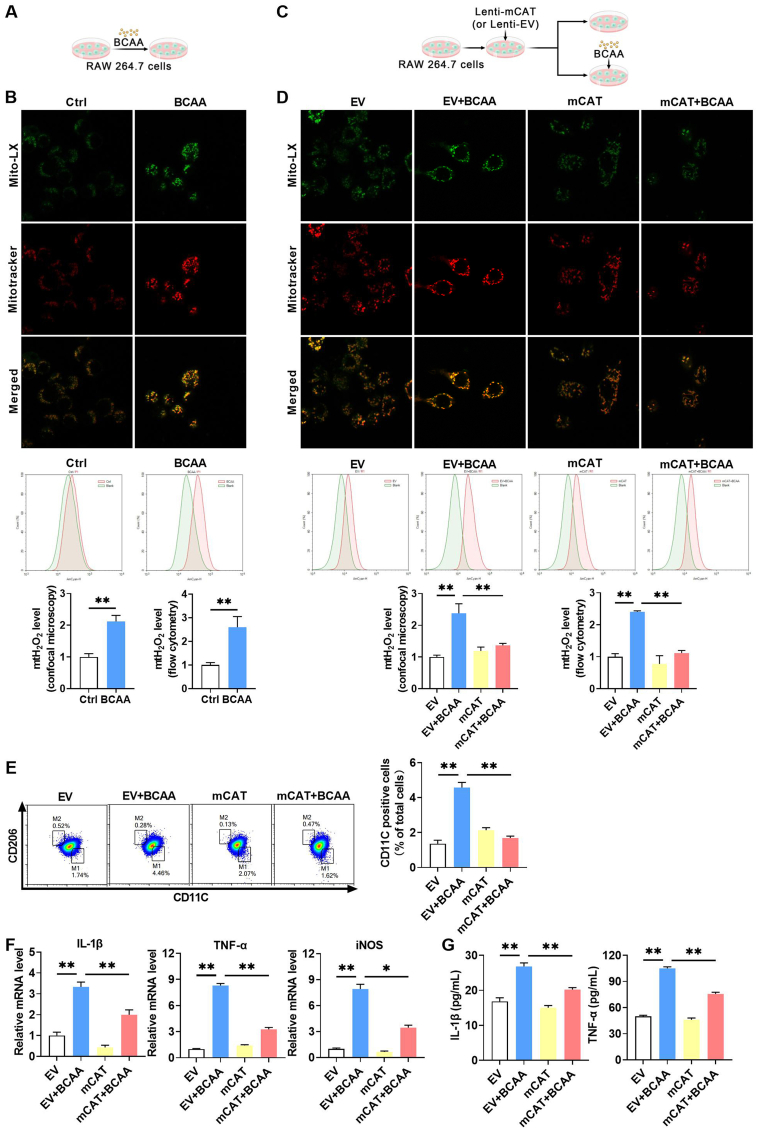
Mitochondrial H_2_O_2_ contributes to BCAA-activated proinflammatory macrophages (A) Schematic diagram of experimental procedure for RAW 264.7 macrophages. (B)Mitochondrial H_2_O_2_ level in RAW 264.7 macrophages by confocal microscopy and flow cytometry. (C) Schematic diagram of experimental procedure for RAW 264.7 macrophages with mCAT overexpression. (D) Mitochondrial H_2_O_2_ level in RAW 264.7 macrophages with mCAT overexpression by confocal microscopy and flow cytometry. (E) CD11C positive cells in RAW 264.7 macrophages with mCAT overexpression. (F) Expression levels of proinflammatory macrophage marker in RAW 264.7 macrophages with mCAT overexpression. (G) IL-1β and TNF-α levels in RAW 264.7 macrophages with mCAT overexpression. Data were expressed as mean ± SEM of three independent experiments. B: data were analyzed with unpaired Student *t*-test; D–G: data were analyzed with one-way ANOVA followed by Tukey's multiple comparisons test. *P < 0.05; **P < 0.01.

Previous studies, including ours, have indicated that elevated BCAA increased reactive oxygen species (ROS) in cardiomyocytes [5,29]. Importantly, the mitochondria are important organelles that not only catabolize BCAA but also produce ROS [1,30]. By using mitochondria-targeted two photo fluorogenic H_2_O_2_ probe [22], we found BCAA significantly increased mtH_2_O_2_ level in RAW 264.7 macrophages (Fig. 4B). Of note, mitochondria-targeted catalase (mCAT), which was applied to specifically clear mtH_2_O_2_ (Fig. 4D), significantly decreased the percentage of CD11C positive cells, as well as the expression and secretion of inflammatory cytokines (Fig. 4E–G) in BCAA-incubated macrophages. Taken together, these findings indicated that mtH_2_O_2_ mediated BCAA-activated proinflammatory macrophages.

### 3.5 HMGB1 signaling mediates the proinflammatory macrophages stimulated by BCAA

**Fig. 5:**
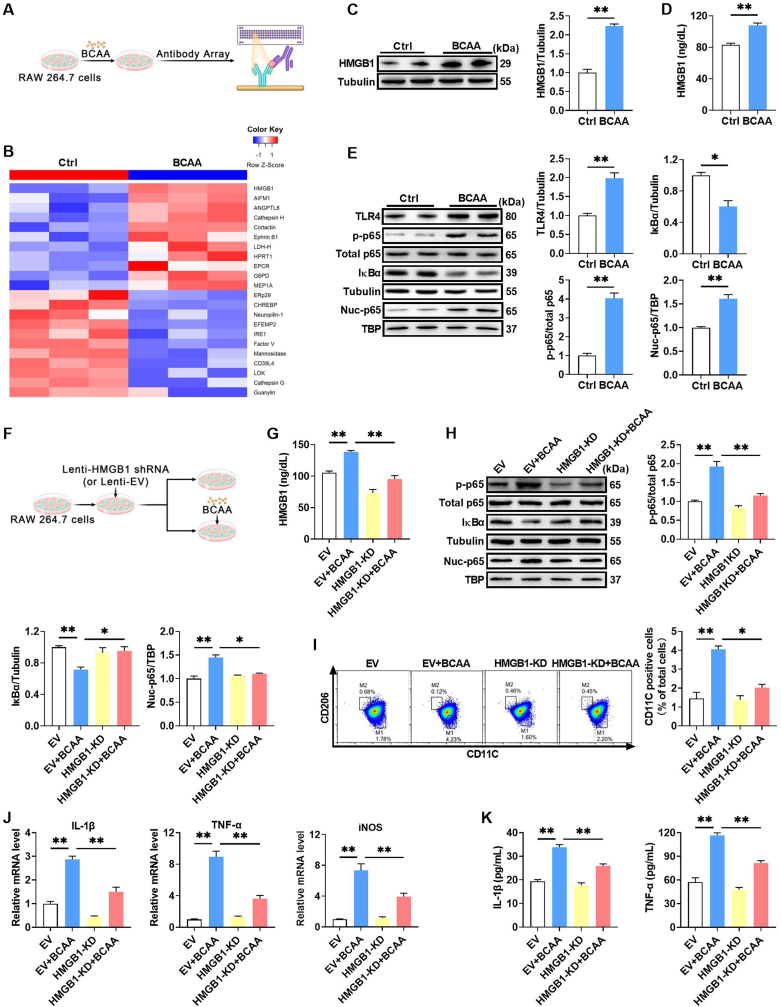

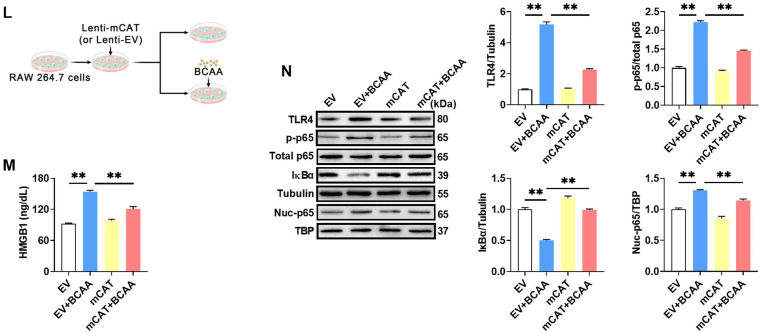
HMGB1 signaling mediates the proinflammatory macrophages stimulated by BCAA (A) Schematic diagram of experimental procedure. (B) Inflammatory cytokines screening by antibody array assay. (C) Cellular HMGB1 level in RAW 264.7 macrophages. (D) Extracellular HMGB1 level in RAW 264.7 macrophages. (E) Protein levels of TLR4, p65, p-p65, IκBα and nuclear p65 in RAW 264.7 macrophages. (F) Schematic diagram of experimental procedure HMGB1-KD RAW 264.7 macrophages. (G) Extracellular HMGB1 level in HMGB1-KD RAW 264.7 macrophages. (H) Expression levels of p65, p-p65, IκBα and nuclear p65 in HMGB1-KD RAW 264.7 macrophages. (I) CD11C positive cells in HMGB1-KD RAW 264.7 macrophages. (J) Expression levels of proinflammatory macrophage markers in HMGB1-KD RAW 264.7 macrophages. (K) IL-1β and TNF-α levels in HMGB1-KD RAW 264.7 macrophages. (L) Schematic diagram of experimental procedure for RAW 264.7 macrophages with mCAT overexpression. (M) Extracellular HMGB1 level in RAW 264.7 macrophages with mCAT overexpression. (N) Protein levels of TLR4, p65, p-p65, IκBα and nuclear p65 in RAW 264.7 macrophages with mCAT overexpression. Data were expressed as mean ± SEM of three independent experiments. B–E: data were analyzed with unpaired Student *t*-test; G–N: data were analyzed with one-way ANOVA followed by Tukey's multiple comparisons test. *P < 0.05; **P < 0.01.

To specify the mechanisms underlying the effects of BCAA-mtH_2_O_2_ on proinflammatory macrophage activation, we conducted protein screening assay (Fig. 5A). As shown in Fig. 5B, of the 500 proteins, 22 differentially expressed proteins were identified in BCAA-treated RAW 264.7 macrophages, including 11 downregulated proteins and 11 upregulated proteins. Notably, among these significantly changed proteins, HMGB1 plays a critical role in inflammation [31]. Western blotting also verified that BCAA greatly increased HMGB1 expression in macrophages (Fig. 5C). Upon stimuli, HMGB1, the conserved nuclear protein, is secreted into the extracellular space to bind TLR4, leading to the activation of proinflammatory macrophages through NF-κB pathway [11,12]. In the present study, BCAA markedly increased HMGB1 secretion of macrophages (Fig. 5D). P65, the core component of NF-κB, normally exists in an inactive cytoplasmic complexes bounded by members of the inhibitor of κB (IκB) family, such as IκBα [32]. After activation, IκBα is gradually degraded and p65 translocates to nucleus, where it binds specific sites in DNA to regulate the expression of inflammatory cytokines [33,34]. In the presence of BCAA, the levels of TLR4, p-p65 and nuclear p65 markedly increased while the level of cytoplasmic IκBα significantly decreased (Fig. 5E), coupled with the activation of proinflammatory macrophages (Fig. 3Q–S), indicating the initiation of TLR4/NF-κB pathway by BCAA. Knockdown of HMGB1 in macrophages (HMGB1-KD, Fig. 5F, Figs. S3C and S3D) markedly alleviated the effects of BCAA on the activation of TLR4/NF-κB pathway and subsequently the generation of inflammatory cytokines (Fig. 5G–K). Together, these findings validated the necessity of HMGB1 signaling in the activation of proinflammatory macrophages by BCAA. Considering that the mtH_2_O_2_ mediated the BCAA-induced activation of macrophages, as shown in Fig. 4, we further evaluated the role of mtH_2_O_2_ on HMGB1 activation (Fig. 5L). We found that scavenging mtH_2_O_2_ by mCAT significantly blocked HMGB1 secretion and activation of TLR4/NF-κB pathway by BCAA (Fig. 5M and N). The above findings suggested that HMGB1 mediated the activation of proinflammatory macrophages by BCAA-induced mtH_2_O_2_.

### 3.6 BCAA regulates disulfide HMGB1 secretion via mitochondrial-to-nuclear H_2_O_2_

**Fig. 6:**
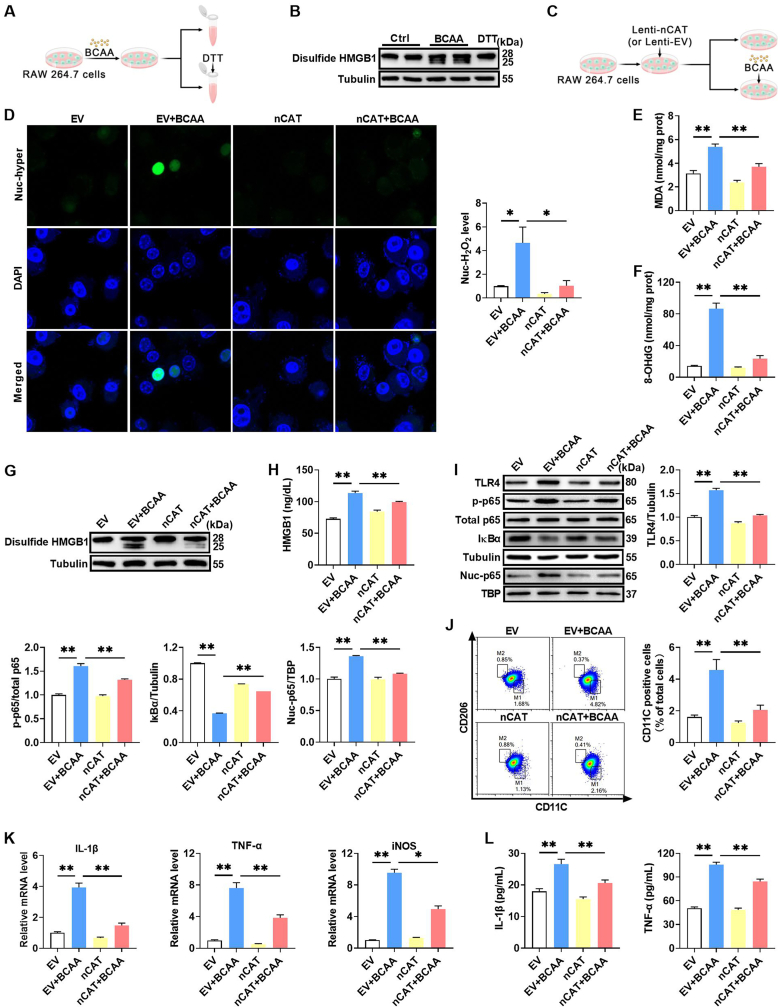

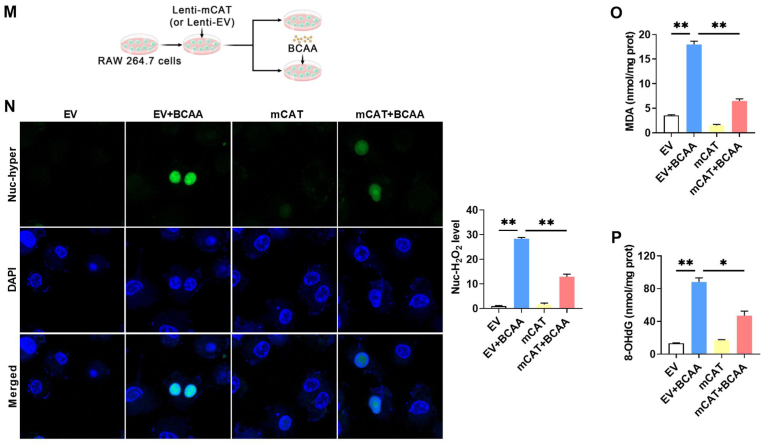
BCAA stimulates disulfide HMGB1 secretion via mitochondria-to-nuclear H_2_O_2_ (A) Schematic diagram of experimental procedure. (B) The level of disulfide HMGB1 in RAW 264.7 macrophages. (C) Schematic diagram of experimental procedure for RAW 264.7 macrophages with nCAT overexpression. (D) Nuclear H_2_O_2_ level in RAW 264.7 macrophages with nCAT overexpression. (E) Nuclear MDA level in RAW 264.7 macrophages with nCAT overexpression. (F) Nuclear 8-OHdG level in RAW 264.7 macrophages with nCAT overexpression. (G) The level of disulfide HMGB1 in RAW 264.7 macrophages with nCAT overexpression. (H) Extracellular HMGB1 level in RAW 264.7 macrophages with nCAT overexpression. (I) Protein levels of TLR4, p65, p-p65, IκBα and nuclear p65 in RAW 264.7 macrophages with nCAT overexpression. (J) CD11C positive cells in RAW 264.7 macrophages with nCAT overexpression. (K) Expression levels of proinflammatory macrophage markers in RAW 264.7 macrophages with nCAT overexpression. (L) IL-1β and TNF-α levels in RAW 264.7 macrophages with nCAT overexpression. (M) Schematic diagram of experimental procedure for RAW 264.7 macrophages with mCAT overexpression. (N) Nuclear H_2_O_2_ level in RAW 264.7 macrophages with mCAT overexpression. (O) Nuclear MDA level in RAW 264.7 macrophages with mCAT overexpression. (P) Nuclear 8-OHdG level in RAW 264.7 macrophages with nCAT overexpression. Data were expressed as mean ± SEM of three independent experiments. All data were analyzed with one-way ANOVA followed by Tukey's multiple comparisons test. *P < 0.05; **P < 0.01.

HMGB1 undergoes extensive post-translational modifications (PTMs) which increase its cytoplasmic translocation and secretion during cell stress [35]. Cysteine thiols are the most reactive functional groups in HMGB1, and their disulfide linkages is a common PTM in proteins entering the secretory pathway [36]. We found that BCAA markedly increased the amount of disulfide HMGB1 in macrophages (Fig. 6B). Studies have reported that exogenous H_2_O_2_ significantly increases the formation and secretion of disulfide HMGB1 [37]. In the present study, BCAA significantly increased nuclear H_2_O_2_ in macrophages, as detected by Nu-HyPer fluorescence, and substantially increased the generation of oxidative stress markers, including MDA and 8-OHdG. Furthermore, we overexpressed a nucleus-targeted calatase (nCAT) to specifically scavenge nuclear H_2_O_2_ in macrophages. This nCAT overexpression markedly attenuated BCAA-induced nuclear oxidative stress (Fig. 6D–F), constrained the formation and secretion of disulfide HMGB1, and it inhibited the activation of NF-κB pathway and proinflammatory macrophages, along with the suppressed inflammatory cytokines production (Fig. 6G-L). These findings demonstrated that BCAA promoted the formation of disulfide HMGB1 and proinflammatory cascade downstream in a nuclear H_2_O_2_ dependent way.

To elucidate the relationship between mtH_2_O_2_ and nuclear H_2_O_2_ in the presence of BCAA, we evaluated the effect of mCAT overexpression on nuclear H_2_O_2_ (Fig. 6M). MCAT overexpression significantly mitigated BCAA-induced nuclear H_2_O_2_ accumulation, as well as MDA and 8-OHdG production (Fig. 6N-P), indicating mtH_2_O_2_ as a crucial signaling molecule which induces nuclear H_2_O_2_ by BCAA. All the aforementioned results suggested that BCAA induced the activation of HMGB1-regulated proinflammatory macrophages via mitochondrial-to-nuclear H_2_O_2_ signaling pathway.

### 3.7 Improving BCAA catabolism of macrophages alleviates AS burden

**Fig. 7:**
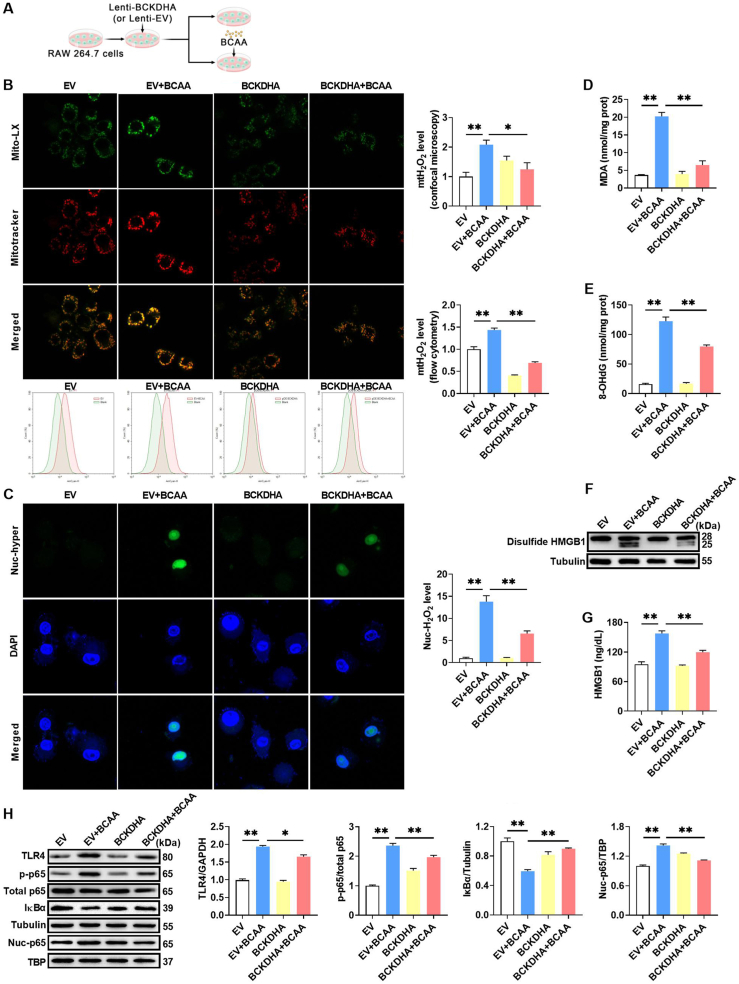

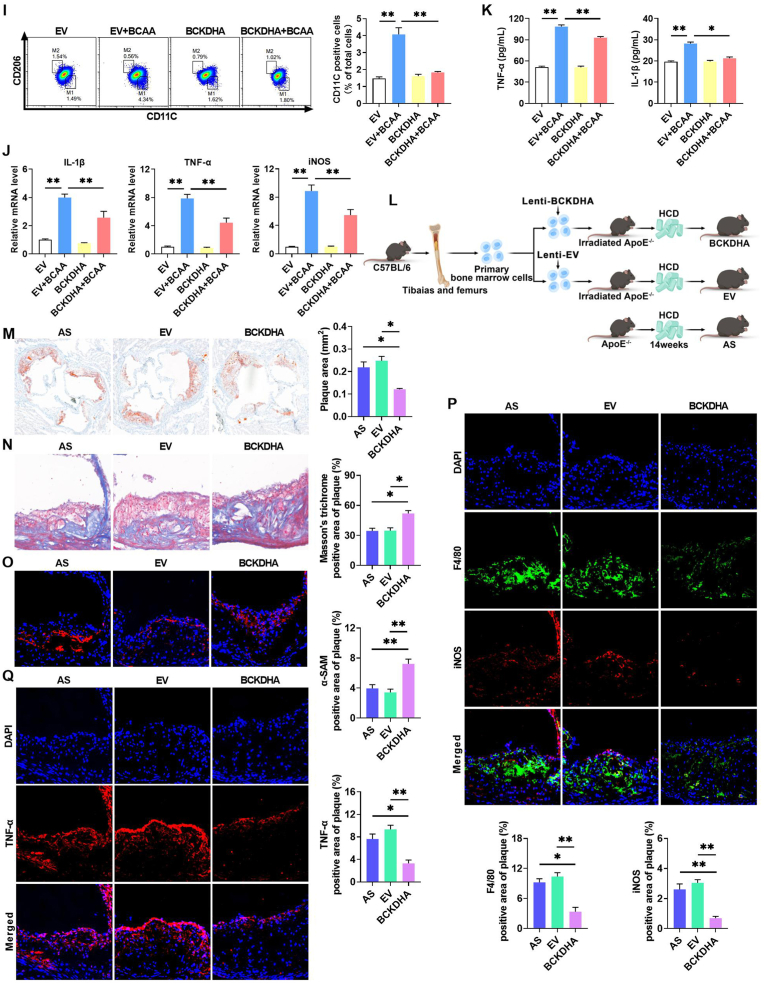
Improving BCAA catabolism of macrophages alleviates AS burden (A) Schematic diagram of experimental procedure for RAW 264.7 macrophages with BCKDHA overexpression. (B) Mitochondrial H_2_O_2_ level in RAW 264.7 macrophages with BCKDHA overexpression by confocal microscopy and flow cytometry. (C) Nuclear H_2_O_2_ level in RAW 264.7 macrophages with BCKDHA overexpression by confocal microscopy. (D) Nuclear MDA level in RAW 264.7 macrophages with BCKDHA overexpression. (E) Nuclear 8-OHdG level in RAW 264.7 macrophages with BCKDHA overexpression. (F) Cellular disulfide HMGB1 in RAW 264.7 macrophages with BCKDHA overexpression. (G) Extracellular HMGB1 level in RAW 264.7 macrophages with BCKDHA overexpression. (H) Protein levels of TLR4, p65, p-p65, IκBα and nuclear p65 in RAW 264.7 macrophages with BCKDHA overexpression. (I) CD11C positive cells in RAW 264.7 macrophage with BCKDHA overexpression. (J) Expression levels of proinflammatory macrophage markers in RAW 264.7 macrophages with BCKDHA overexpression. (K) IL-1β and TNF-α levels in RAW 264.7 macrophages with BCKDHA overexpression. (L) Schematic diagram of animal study with the bone marrow transplantation. (M) Oil Red O staining of the plaque area. (N) Masson's trichrome staining of the collagen content. (O) Alpha-smooth muscle actin (α-SMA) positive cells by immunofluorescence staining showing SMCs. (P) F4/80 and iNOS positive cells by immunofluorescence staining showing macrophages. (Q) TNF-α positive cells by immunofluorescence staining showing the activation of proinflammatory macrophages. B–J: Data were expressed as mean ± SEM of three independent experiments. M–Q: Data were expressed as mean ± SEM, n = 6. All data were analyzed with one-way ANOVA followed by Tukey's multiple comparisons test. *P < 0.05; **P < 0.01. (For interpretation of the references to colour in this figure legend, the reader is referred to the Web version of this article.)

To identify whether accelerating BCAA catabolism might inhibit the activation of proinflammatory macrophages, we overexpressed BCKDHA to facilitate BCAA catabolism in macrophages (Fig. 7A, Figs. S3E and S3F). The overexpression of BCKDHA greatly attenuated the BCAA-induced accumulation of mitochondrial-to-nuclear H_2_O_2_ (Fig. 7B and C) in macrophages, which was accompanied by the markedly decreased nuclear oxidative stress (Fig. 7D and E). Additionally, the overexpression of BCKDHA reversed the effects of BCAA on HMGB1 secretion, TLR4/NF-κB pathway activation, proinflammatory macrophage activation, and inflammatory cytokines release in macrophages (Fig. 7F–K). These findings demonstrated that improving BCAA catabolism attenuated the activation of proinflammatory macrophages.

As macrophages originate primarily from myeloid progenitor cells in the bone marrow [38], we further transplanted bone marrow cells with BCKDHA overexpression (Figs. S3G and S3H) into AS mice (Fig. 7L) to specifically improve BCAA catabolism of macrophages in vivo. The transplantation of BCKDHA-overexpressing bone marrow cells significantly alleviated the AS burden in mice, as evidenced by the decreased plaque volume along with the increased plaque stability (increased collagen content and smooth muscle cells) (Fig. 7M − O) as well as notable decreases of F4/80, iNOS and TNF-α positive areas in the plaques (Fig. 7P and Q). Together, the above results suggested that improving BCAA catabolism of macrophages alleviated AS progression and inflammation.

## 4 Discussion

The role of glucose and lipid metabolisms in AS and its related diseases have been well documented, whereas the relationship of amino acids with AS remains unknown. This study revealed that elevated BCAA level was an independent risk factor for CHD. Elevated BCAA due to high dietary BCAA intake or BCAA catabolic defects promoted AS progression, while the improved BCAA catabolism alleviated AS. BCAA activated proinflammatory macrophages by increasing mtH_2_O_2_, which eventually deteriorated the atherosclerotic inflammation. Explicitly, BCAA induced disulfide HMGB1 formation and secretion in macrophages via mitochondrial-nuclear H_2_O_2_ signaling, which activated proinflammatory macrophages and triggered the inflammation through HMGB1/TLR4/NF-κB pathway.

As the essential amino acids derived from diet, BCAA is beneficial to maintaining the body normal physical functions. BCAA supplementation has been recommended for the elderly, the patients with consumptive diseases and athletes [1,2]. However, recent studies, consistent to ours, suggest an association between elevated BCAA level with greater risk of diseases [3,4]. Hiraiwa et al. showed that the ratio of plasma BCAA to aromatic amino acids can predict future cardiac events in patients with heart failure [39,40]. Kubacka et al. reported that elevated BCAA level was associated with cardiometabolic risk factors with the cutoff value of 475.0 μmol/L in women [41]. Our study found that plasma BCAA level was an independent risk factor for CHD and men with plasma BCAA level greater than 63.96 mg/L (∼505.61 μmol/L) were more likely to have CHD. Yet, large-scale randomized trials are still needed to validate our findings in the future. It is also observed that the supplementation of BCAA remarkably promoted AS progression in an AS model of ApoE^−/−^ mice, while reduction of BCAA by enhancing their catabolism attenuated the AS burden, demonstrating the critical role of BCAA in AS development. Moreover, our study first verified that the elevated BCAA did not affect the plasma glucose and lipid levels, indicating that BCAA may promote AS progression independent to glucose and lipid metabolism. Apart from dietary intake, BCAA level is mainly determined by their catabolism. Interestingly, BCAA catabolic defects were observed in both CHD patients and AS mice, suggesting a potential genetic susceptibility of BCAA-induced AS related diseases. Restriction of BCAA-rich food intake, especially for the people with BCAA catabolic defects may be a novel strategy for AS prevention, and targeting BCAA catabolic defects may be a promising therapeutic approach to AS and CHD management. Additionally, previous literatures have reported the critical role of BCAA individuals in diseases. Yu et al. reported that isoleucine and valine were key regulators of metabolic health and the adverse metabolic response to dietary BCAA, such as obesity and diabetes [42]. Xu et al. reported that valine played a major role in BCAA-facilitated platelet activation [6], and Jang et al. found that 3-HIB, a catabolic intermediate of the BCAA valine, promoted insulin resistance [43]. In our present study, the OR value of the individuals also suggested that the valine was more important in the correlation of BCAA with CHD. This is great important and further studies are warranted in our future work.

Monocyte-macrophages-induced inflammation contributes to all stages of AS. Zhenyukh et al. reported that exogenous BCAA induces inflammation in endothelial cells by activating peripheral blood mononuclear cells [15]. Liu et al. found that BCKA promotes cytokine production of diabetic bone marrow-derived macrophages [44]. In the present study, we observed that increased BCAA level, which may due to catabolic defects in circulating monocytes from patients with CHD and abdominal macrophages from AS mice, were accompanied with activation of proinflammatory macrophages and production of inflammatory cytokines. The induction of BCAA on macrophages was further verified by interventions of exogenous BCAA supplementation and endogenous BCAA catabolic enhancement by BCKDHA-KD. Moreover, in vivo transplantation of bone marrow cells overexpressing BCKDHA into AS mice to specifically improve BCAA catabolism in macrophages, not only inhibited the inflammation by macrophages in atherosclerotic plaques, but also decreased plaque volume and instability of AS. We thereby conclude that BCAA enhanced atherosclerotic inflammation via activating proinflammatory macrophages, a critical step in AS. As we know, liver, skeletal muscle, adipose and heart are important tissues and organs for BCAA metabolism [1,45,46]. It is possible that the defect of BCAA metabolism in these tissues and organs lead to the accumulation of plasma BCAA, which further promote AS through activation of proinflammatory macrophages. Elucidating the target organ responsible for the elevated BCAA level should be great important in our future study.

BCAA is mainly catabolized in mitochondria [1,47], and coincidentally the mitochondrial respiratory chain is also the main source of ROS [30]. Our previous study has found that exogenous BCAA stimulated the ROS production in the cardiac tissues of diabetic mice [29]. Normally, the electrons leaked from mitochondrial respiratory chain are transported to molecular oxygen to produce superoxide union (O_2_^•−^) with a half-live of only about 10^−6^ Sec at 37 °C [48]. O_2_^•−^ is then converted to hydrogen peroxide (H_2_O_2_) by superoxide dismutases (SODs) rapidly, which is further catalyzed to H_2_O and O_2_ by catalase (CAT). As such, H_2_O_2_ is recognized as the major ROS in redox signaling regulation [49]. In the present study, BCAA supplementation significantly stimulated the mtH_2_O_2_ production in macrophages. Specifically, scavenging mtH_2_O_2_ by mCAT virtually offset the activation of macrophages and the production of inflammatory cytokines, suggesting that mtH_2_O_2_ is essential in mediating the activation of macrophages by BCAA.

In order to probe into the underlying molecular mechanism for macrophages activation, we identified HMGB1 as the potential target molecule of BCAA-mtH_2_O_2_ by the protein screening assay. Previous studies indicated that extracellular HMGB1 is involved in accelerating macrophage polarization toward M1 phenotype [50,51]. In the present study, BCAA supplementation significantly stimulated HMGB1 secretion and an inflammatory cascade downstream via TLR4/NF-κB pathway, on which knockdown of HMGB1 showed remarkable inhibition, figuring out HMGB1 as indispensable for BCAA-mtH_2_O_2-_induced inflammation.

Question remains as to how BCAA-induced mtH_2_O_2_ stimulates HMGB1 secretion at this stage. HMGB1 is a highly conserved nuclear protein consisting of three conserved cysteine residues: Cys23, Cys45 and Cys106 [37]. It is reported that redox modifications of HMGB1 such as the disulfide bond between Cys23 and Cys45 can increase HMGB1 cytoplasmic accumulation and extracellular secretion in response to inflammatory stimuli [37]. Our results showed that BCAA treatment promoted the formation and secretion of disulfide HMGB1, thereby further activating an inflammatory cascade in macrophages. As HMGB1 normally exist in nucleus, we speculated that BCAA treatment could induce oxidative stress in nucleus. As expected, we found that both nuclear H_2_O_2_ (nH_2_O_2_) and oxidative products MDA and 8-OHdG increased after BCAA treatment. Specifically, scavenging nH_2_O_2_ by nCAT significantly inhibited the formation and secretion of disulfide HMGB1, as well as the activation of macrophages and the production of inflammatory cytokines. Moreover, mitochondria and nucleus cross-talk has been found in diversified ways including ROS [52]. We also found that scavenging mtH_2_O_2_ remarkably reduced nH_2_O_2_ induced by BCAA. Future research will be required to determine if mtH_2_O_2_ directly diffused into the nucleus or instead it stimulated the nucleus to produce H_2_O_2_. Together, our results showed that BCAA caused the inflammatory cascade of macrophages and the formation and secretion of disulfide HMGB1 in a mitochondrial-nuclear H_2_O_2_ dependent manner, and thus triggered the progression of AS.

**Fig. 8:**
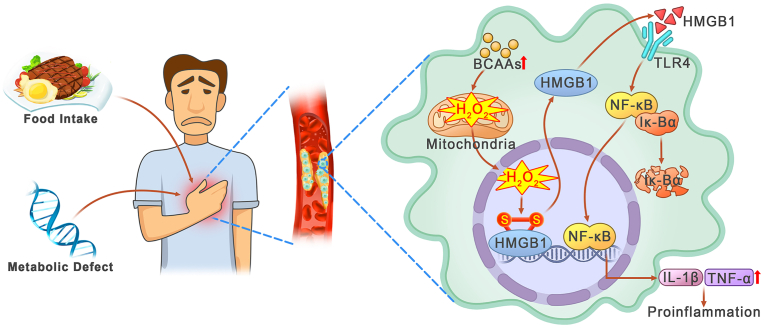
Schematic of BCAA promoted AS by enhancing mitochondrial-to-nuclear H_2_O_2_-disulfide HMGB1 in macrophages

In conclusion, we revealed elevated BCAA level as an independent risk factor for CHD. Elevated BCAA due to high dietary BCAA intake or BCAA catabolic defects promoted the AS progression, while improved BCAA catabolism alleviated AS. Moreover, we uncovered a previously unknown mechanism wherein BCAA caused HMGB1 to be modified and secreted in a disulfide form via mitochondrial-nuclear H_2_O_2_, which in turn activated inflammatory macrophages, contributing to the AS and subsequent CHD (Fig. 8). These findings provide new insights into the role of animo acids as the daily dietary nutrients in AS development, and also suggest that restricting excessive dietary BCAA intake and promoting BCAA catabolism may be effective strategies for prevention and treatment of AS and its related diseases.

## Author contributions

S.Z., L.Z., Q.W., and J-H.C. performed the experiments. Y.C., B-D.Z., and Z-H.W. helped on the animal studies. C-Y.L., G-Y.Z., and R.L helped on the in vitro studies. W.W., Z-J.T., and H-P.Z. analyzed the data. K.L., X-J.Q. and H-K.G. designed the experiments and wrote the paper. C-X.L supervised the study and critically reviewed the paper.

## Declaration of competing interest

The authors declare no competing interests.

## Data Availability

Data will be made available on request.
