# Supplementary material for: Elevated branched-chain amino acid promotes atherosclerosis progression by enhancing mitochondrial-to-nuclear H2O2-disulfide HMGB1 in macrophages
Source: Redox Biol. 2023 Apr 5;62:102696. doi: 10.1016/j.redox.2023.102696 (PMC10130699; doi:10.1016/j.redox.2023.102696)
Supplement: Multimedia component 1 [file mmc1.docx]

**Supplementary Materials**

**Figure S1**


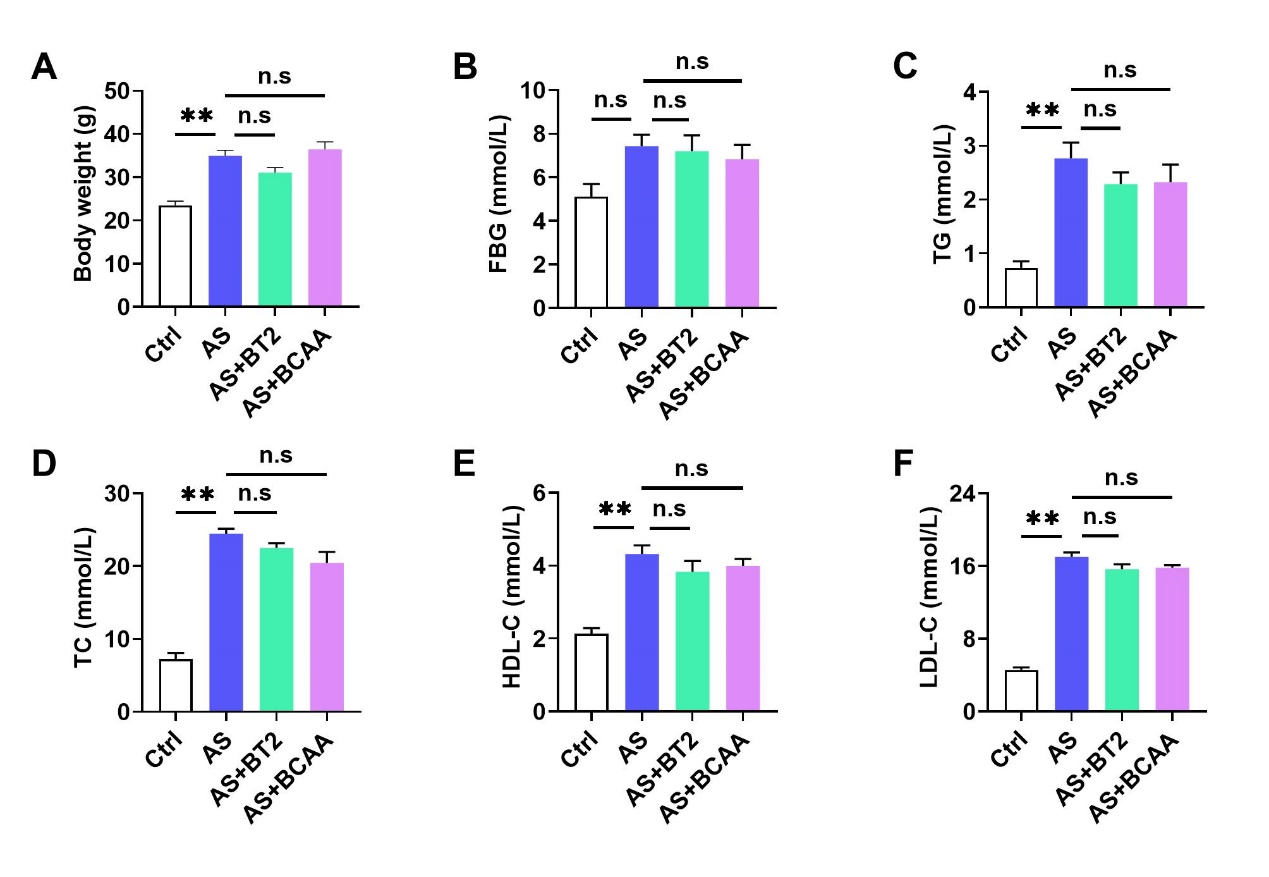


**Figure S1. Elevated BCAA has no obvious effect on body weight and glycolipid levels in HCD-fed ApoE^-/-^ Mice**

(A) Body weight of mice. (B) Plasma fasting blood glucose (FBG) level in mice. (C-F) Plasma lipid parameters in mice. Data were expressed as mean ± SEM, n=5. All data were analyzed with one-way ANOVA followed by Tukey's multiple comparisons test. ^**^*P*<0.01; n.s, not significant.

**Figure S2**


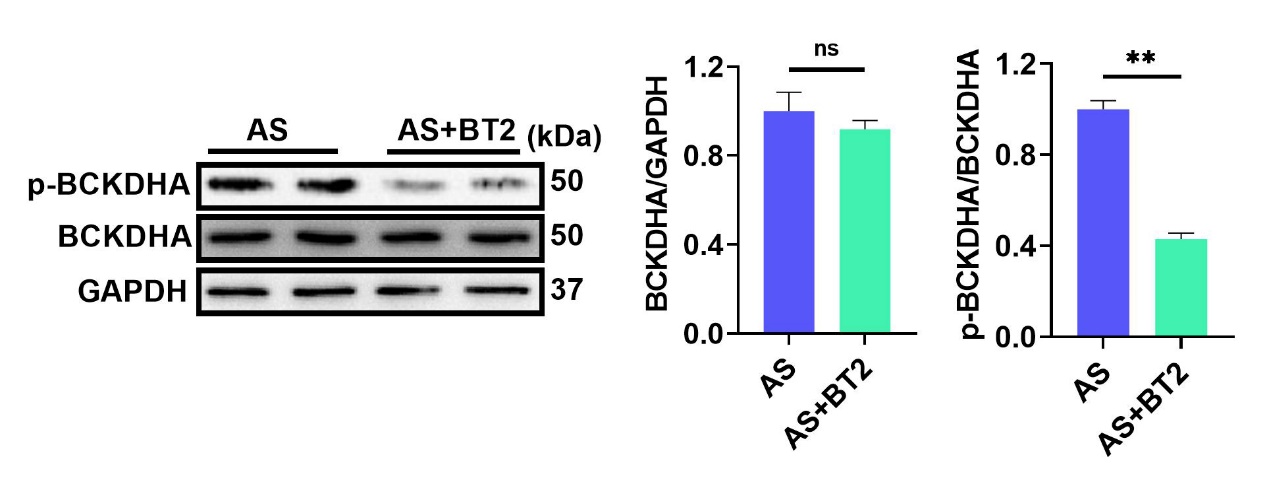


**Figure S2. BT2 treatment significantly activates BCKDHA in liver.**

Protein levels of BCKDHA and p-BCKDHA/BCKDHA in liver. Data were expressed as mean ± SEM, n=3. All data were analyzed with unpaired Student *t* test. n.s, not significant; ^**^*P*<0.01.

**Figure S3**

**
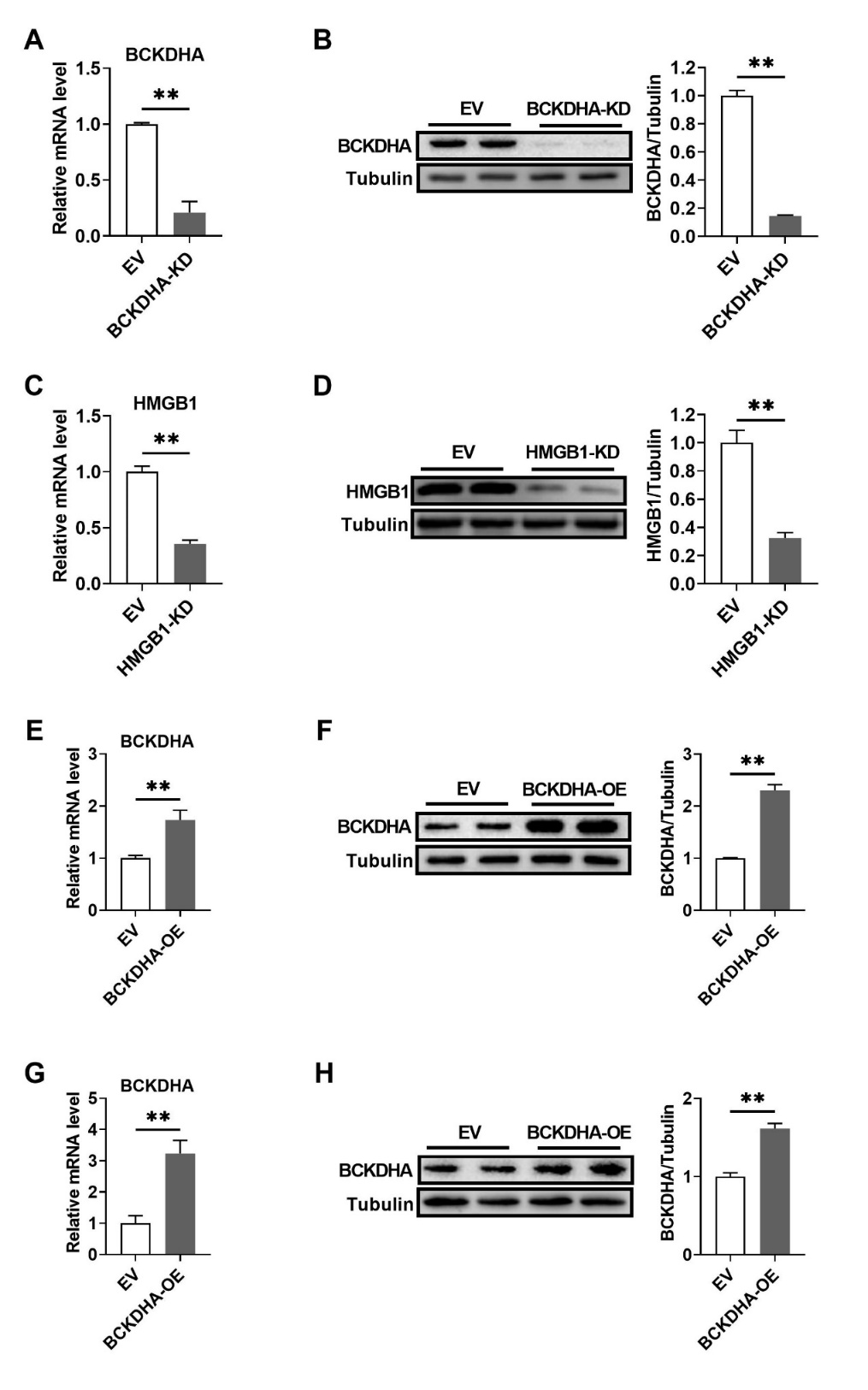
**

**Figure S3. Genetic intervention targeting BCKDHA or HMGB1 in RAW 264.7 macrophages or ApoE**^-/-^ **mice**

(A)-(B) Expression level of BCKDHA in BCKDHA-KD RAW 264.7 macrophage. (C)-(D) Expression level of HMGB1 in HMGB1-KD RAW 264.7 macrophages. (E)-(F) Expression level of BCKDHA in BCKDHA-overexpressed RAW 264.7 macrophages. (G)-(H) Expression level of BCKDHA in BCKDHA-overexpressed ApoE^-/-^ mice by bone marrow transplantation. Data were expressed as mean ± SEM of three independent experiments. All data were analyzed with unpaired Student *t* test. ^**^*P* < 0.01.

**Table S1. Correlation between plasma BCAA and glycolipid levels in CHD patients**

| **Factors** | **Correlation (*P*)** |
| --- | --- |
| **FBG** | -0.025(0.706) |
| **TC** | 0.033(0.616) |
| **TG** | 0.085(0.196) |
| **LDL-C** | 0.052(0.431) |
| **HDL-C** | -0.046(0.483) |

BCAA, branched-chain amino acid; FBG, fasting blood glucose; TC, total cholesterol; TG, triglyceride; LDL-C, low density lipoprotein cholesterol; HDL-C, high density lipoprotein cholesterol.

**Table S2. Univariate and multivariate logistic regression analysis for discrimination of CHD.**

|  | **Univariate Analysis** | | | | **Multivariate Analysis** | | | |
| --- | --- | --- | --- | --- | --- | --- | --- | --- |
|  | **OR** | **95%CI** | **P Value** | **Standardized**  **OR** | **OR** | **95%CI** | **P**  **Value** | **Standardized**  **OR** |
| **BCAA** | 1.089 | 1.063-1115 | <0.01 | 5.183 | 1.076 | 1.037-1.134 | <0.01 | 4.133 |
| **Leu** | 1.200 | 10129-1.276 | <0.01 | 3.176 | 1.177 | 1.062-1.305 | <0.01 | 2.861 |
| **Ile** | 1.304 | 1.189-1.429 | <0.01 | 2.935 | 1.247 | 1.069-1.455 | <0.01 | 2.478 |
| **Val** | 1.226 | 1.162-1.293 | <0.01 | 7.175 | 1.186 | 1.097-1.282 | <0.01 | 5.418 |

Age, BMI, TG, TC, LDL-C, HDL-C and total BCAA or individual BCAA were included in multivariate logistic regression analysis. Leu, leucine; Ile, isoleucine; Val, valine

**Table S3. q-PCR primer sequences**

| **Target gene** | **Primer sequence (forward)** | **Primer sequence (reverse)** |
| --- | --- | --- |
| **Human BCAT2** | AAGGCGTTCAAAGGCAAAGACCA | TGTCGAAACTCGGCAGGCACA |
| **Human BCKDHA** | TGCTGAGCCAAGGCTGGTGG | TGGGCGGGCATCTCCTGATA |
| **Human PP2Cm** | ATAACCGCATTGATGAGCCA | TCCGTTTGCCAATCTGTGAG |
| **Human β-actin** | CACGATGGAGGGGCCGGACTCATC | TAAAGACCTCTATGCCAACACAGT |
| **Mouse BCAT2** | TTCATTCGTCAGAGCCTGGATA | ACTACTCCAGGCAAGATGACGC |
| **Mouse BCKDHA** | TGCTGAGCCAAGGCTGGTGG | TGGGCGGGCATCTCCTGATA |
| **Mouse PP2Cm** | TCTCATTGGCAAACGGAAAG | CAGACAGGTGGGCATAACTCG |
| **Mouse IL-1β** | CTCACAAGCAGAGCACAAGC | ACGGATTCCATGGTGAAGTC |
| **Mouse TNF-α** | CCGATGGGTTGTACCTTGTC | GTGGGTGAGGAGCACGTAGT |
| **Mouse iNOS** | AGTTCCCTTCCTTGCATGTG | GAGTAGTAGCGGGGCTTCAA |
| **Mouse β-actin** | CACGATGGAGGGGCCGGACTCATC | TAAAGACCTCTATGCCAACACAGT |
